# Neurophysiological effects of latent trigger point dry needling on spinal reflexes

**DOI:** 10.1152/jn.00366.2024

**Published:** 2024-12-20

**Authors:** Gretchen Seif, Alan M. Phipps, Joseph M. Donnelly, Blair H. S. Dellenbach, Aiko K. Thompson

**Affiliations:** ^1^Department of Health Professions, College of Health Professions, Medical University of South Carolina, Charleston, South Carolina, United States; ^2^Department of Health Sciences and Research, College of Health Professions, Medical University of South Carolina, Charleston, South Carolina, United States; ^3^Department of Physical Therapy, University of St. Augustine for Health Sciences, Miami, Florida, United States

**Keywords:** H reflex, latent trigger point, medial gastrocnemius, plantarflexor, reciprocal inhibition

## Abstract

Deep dry needling (DDN) is a method to treat muscle trigger points (TrPs) often found in persons with neuromuscular pain and spasticity. Currently, its neurophysiological actions are not well established. Thus, to understand how DDN affects spinal cord physiology, we investigated the effects of TrP DDN on spinal reflexes. In 17 adults with latent TrPs in the medial gastrocnemius (MG) without known neurological or orthopedic injuries, the H reflex, M wave, and reciprocal inhibition in the soleus, MG, and lateral gastrocnemius (LG) and passive ankle range of motion (ROM) were measured before and immediately, 90 min, and 72 h after a single bout of DDN at the MG TrPs. The MG maximum M wave (M_max_) amplitude was decreased immediately and 90 min post DDN (by −14% and −18%) and returned to pre-DDN level at 72 h post. LG and soleus M_max_ did not change. The maximum H reflex (H_max_) amplitude did not change in any of the triceps surae. Soleus inhibition was increased significantly immediately (+30%) and 72 h (+36%) post DDN. ROM was increased by ≈4° immediately and ≈3° at 72 h post DDN. Temporary reduction of MG (but not soleus or LG) M_max_ amplitude after DDN and its recovery at 72 h post indicate temporary and specific effects of DDN in the treated muscle. The immediate and 72 h post increases in the ROM and soleus inhibition with no changes in H_max_ suggest complex effects of DDN at the spinal level.

**NEW & NOTEWORTHY** In this study, we examined the effects of deep dry needling (DDN) on spinal reflexes in the triceps surae. We found that the H reflex (an excitatory reflex) did not change after DDN but soleus inhibition was increased immediately and 72 h after DDN, corresponding to increases in ankle range of motion. Differential effects of DDN on excitatory and inhibitory reflexes over the first 72 h may reflect its complex neurophysiological effects at the spinal level.

## Graphical abstract

**Figure:**
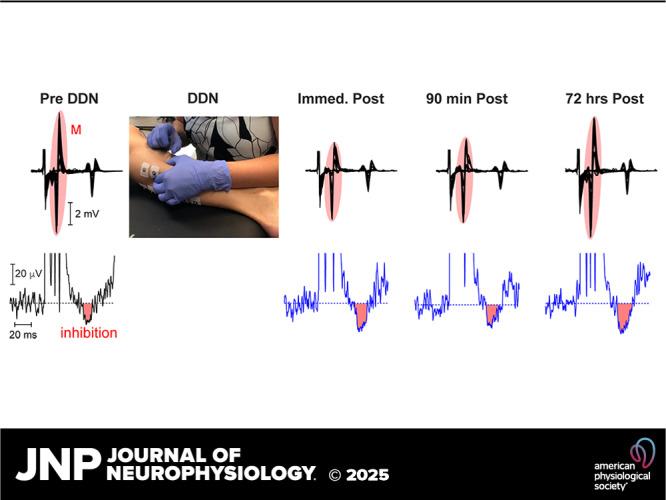

## INTRODUCTION

Trigger point (TrP) deep dry needling (DDN) is becoming a common method to treat active and latent TrPs in persons with pain and, more recently, in persons with spasticity (1–17). Active and latent TrPs are hyperirritable taut bands in a muscle, where palpation can produce tenderness and elicit a local twitch response (LTR) (18). Active TrPs produce referred pain (4), whereas latent TrPs may or may not; however, it is proposed that latent TrPs may alter functioning of the muscle, its agonists, and antagonists (13, 18–22). The presence of latent TrPs may lead to muscle cramping, restricted range of motion (ROM), muscle weakness, altered cocontraction of antagonists, and accelerated fatigability (2, 18–20). Furthermore, the presence of latent TrPs could produce a low-level (subthreshold) nociception, affect the neuronal activity in the dorsal horn of the spinal cord, and increase pain sensitivity (4, 13, 18). Thus, treating latent TrPs could help to reduce sensorimotor discomfort and deficits in people with various musculoskeletal and neuromuscular impairments.

DDN is a method to treat TrPs. It is presumed that DDN of TrPs produces its therapeutic effects through multiple parallel mechanisms. First, it is proposed that DDN of TrPs mechanically disrupts dysfunctional motor end plates by stretching the contracted cytoskeletal structures (i.e., shortened sarcomeres) (23), helping to restore the resting level of sarcomere (i.e., muscle fiber) length. Second, it is thought that DDN excites nociceptors [i.e., A-γ, A-δ, and C (group III/IV) afferents] near TrPs, which in turn influence the excitability of multiple spinal pathways via interneuronal connections (24). This may partly explain how DDN can reduce myofascial pain (11, 12, 14). Third, DDN likely affects the local muscle blood flow and oxygen saturation (3, 25), which presumably helps to reduce pain-producing substances (e.g., bradykinin, calcitonin gene-related peptide, and substance P) that are accumulated at/around TrPs (26, 27). Although these theories provide plausible explanations for how DDN produces therapeutic effects, to date little is understood about how DDN affects the spinal reflex excitability and induces plasticity in the spinal cord pathways (1, 28–32).

Several case studies and recent systematic reviews suggest that in persons post stroke DDN can decrease spasticity, which is commonly thought to contribute to decreased function and quality of life (8, 9, 12, 14–17, 33–35). To establish the clinical utility of DDN, it is essential to better understand its neurophysiological impact on central nervous system (CNS) pathways involved in sensorimotor function. Thus, as the first step in understanding the spinal mechanism of DDN action, in this study we aimed to examine the effects of DDN on inhibitory and excitatory spinal reflex pathways in persons without any neurological conditions or injuries with latent TrPs in the medial gastrocnemius (MG). We examined the effects of applying DDN in one muscle (i.e., MG) on itself and its close synergists lateral gastrocnemius (LG) and soleus, which share heteronymous excitation and inhibition, so that we would have an opportunity to observe the effects that are specific to the treated muscle (i.e., MG) and the effects that are not originated directly from mechanical consequences of DDN (and thus would be of neurophysiological effects of DDN). We chose the MG as the targeted muscle because of the prevalence of TrPs in this muscle (22).

Although they often function synergistically, the gastrocnemii and the soleus are different in many ways. Anatomically, because the gastrocnemii are two-joint muscles whereas the soleus is a single-joint muscle (36), ankle and/or knee joint motion influences their muscle lengths and motor outputs differently. Histochemical properties (i.e., muscle fiber type) differ between the gastrocnemii and the soleus (37–39), which in turn affect their resistance to fatigue (40), contractile properties (41), and electromyographic (EMG) activity during standing and walking (42, 43). In cats, muscle spindle density is greater in the soleus than in the MG (44); excitatory postsynaptic potentials (EPSPs) produced by Ia afferents are larger in the soleus motoneurons than in the MG and LG motoneurons (45, 46) [also true in nonhuman primates (47)]; the three triceps muscles differ in homonymous and heteronymous excitation and inhibition (48); and across the triceps presynaptic inhibition can differ even between collaterals from the same muscle afferent (49). Thus, treating only one of the triceps surae (i.e., MG) while studying all three triceps would create a unique scientific opportunity to examine the presence/absence of potential selective effects of DDN.

Specifically, two spinal pathways were examined in this study: the Hoffmann (or H) reflex and reciprocal inhibition in the triceps surae. The H reflex, elicited by electrical stimulation of a mixed peripheral nerve, is a partial electrical analog of the spinal stretch reflex. Since the soleus H reflex is often large in individuals with spasticity due to chronic spinal cord injury and stroke (50–54), some view the H reflex as a potential tool to evaluate the extent of presence and changes in spastic hyperreflexia (55, 56). Spinal reciprocal inhibition, which can be measured as suppression of ongoing EMG activity induced by antagonist muscle nerve stimulation (57, 58), also becomes abnormal after CNS lesions (50, 53, 59–65). Thus, changes in these spinal reflexes in response to DDN, if occurring, would not only suggest that DDN induces spinal plasticity but also support the possibility that DDN could affect spinal somatosensory processing that is part of abnormal sensorimotor function in people with CNS lesions.

## METHODS

### Participants

Seventeen adults 22–57 yr old (median 27 yr; 11 females and 6 males) without known neurological conditions or orthopedic injuries participated in this study. Before participation, all participants gave written informed consent by themselves, which was reviewed and approved by the Medical University of South Carolina Institutional Review Board. Exclusion criteria were *1*) a cardiac condition (history of myocardial infarction, congestive heart failure, pacemaker use, coronary artery disease, atrial fibrillation, congenital heart disease, uncontrolled hypertension); *2*) a medically unstable condition (including temporary infections and pregnancy); *3*) metal allergies; *4*) needle phobias; *5*) lymphedema over a limb (because of the risk of infection/cellulitis); *6*) abnormal bleeding tendencies; *7*) compromised immune system; *8*) vascular disease; *9*) uncontrolled diabetes; *10*) history of epilepsy (as DDN could generate strong somatosensory sensation); and *11*) anxiety disorders or in distress.

### General Procedures

**Figure 1.**
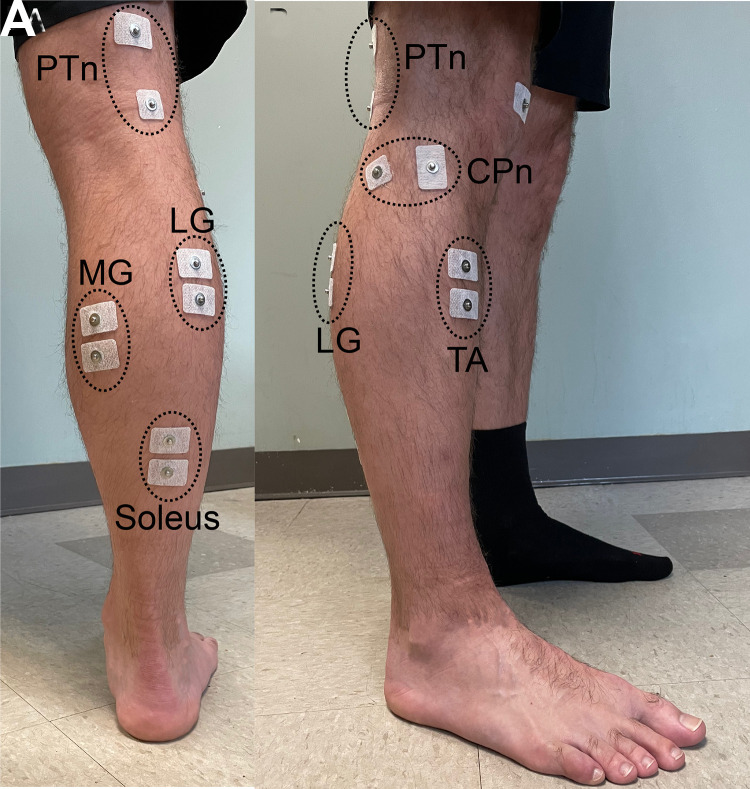
Electromyography (EMG) and nerve stimulating electrode placements. CPn, common peroneal nerve; LG, lateral gastrocnemius; MG, medial gastrocnemius; PTn, posterior tibial nerve; TA, tibialis anterior.

Each participant underwent four sets of experimental measurements that occurred immediately before, immediately after, 90 min after, and 72 h after a single bout of DDN of latent TrPs in the MG. At the beginning of each set (time point) of measurements, electromyography (EMG) recording electrodes were placed over the triceps surae [soleus, medial gastrocnemius (MG), and lateral gastrocnemius (LG)] and tibialis anterior (TA), and nerve stimulating electrodes were placed over the posterior tibial nerve (PTn) and common peroneal nerve (CPn) on the studied leg (Fig. 1). Then, while the participant stood and maintained a preset level (i.e., a natural standing level) of soleus and TA background EMG activity (see *EMG recording and nerve stimulation*), the PTn was stimulated to measure the maximum H reflex (H_max_) and the maximum M wave (M_max_) in the triceps surae. After PTn stimulation, the CPn was stimulated to elicit the M wave in the TA and reciprocal inhibition in the triceps surae while the standing participant maintained the above-mentioned preset level of background EMG activity. At each measurement time point, passive ankle dorsiflexion (DF) range of motion (ROM) was also measured in supine position with the knee extended, with a standard goniometer.

#### EMG recording and nerve stimulation.

EMG signals were continuously recorded from the triceps surae and TA, ipsilateral to the stimulation and DDN site, with pairs of self-adhesive surface Ag-AgCl electrodes (2.2 × 3.3 cm; Vermed/Nissha Medical Technologies, Buffalo, NY) (Fig. 1) with their centers ∼3 cm apart. TA, MG, and LG electrodes were placed over the muscle belly and soleus electrodes placed in line with the Achilles tendon below the gastrocnemius. EMG signals were amplified, band-pass filtered (10–1,000 Hz), sampled at 4,000 Hz, and stored.

To elicit the triceps surae H reflex and M wave the PTn was stimulated and to elicit the TA M wave the CPn was stimulated with Ag-AgCl electrodes (2.2 × 2.2 cm for the cathode and 2.2 × 3.3 cm for the anode; Vermed). PTn stimulation was delivered as single square pulses (1 ms in pulse width) through a Digitimer DS7A stimulator (Digitimer Limited, Letchworth Garden City, UK). PTn stimulation electrodes were placed in the popliteal fossa so as to reduce the soleus, MG, and LG H reflex thresholds, maximize their M_max_ amplitudes, and minimize stimulation of other nerves. CPn stimulation was delivered as a single square pulse (0.5-ms pulse width) through a Digitimer DS7A stimulator and a pair of electrodes placed around the neck of the fibula. For both PTn and CPn stimulation, a stimulus pulse was delivered when the participant had maintained a natural standing level of soleus EMG activity (typically around 24 µV) and resting level of TA EMG (<8 µV) for at least 2 s. The minimum interstimulus interval was 5 s. In the triceps surae, the H reflex-M wave recruitment curves were obtained by gradually increasing the stimulus intensity from below H reflex threshold to the maximum H reflex (H_max_) to beyond an intensity required to elicit the maximum M wave (M_max_) in each of the triceps surae muscles (58, 66–70). Similarly for the TA, the recruitment curve was obtained by gradually increasing the stimulus intensity from below M wave threshold to beyond an intensity required to achieve the M_max_ in the TA. For both the triceps and TA recruitment curves, four responses were averaged at each intensity.

#### Deep dry needling.

**Figure 2.**
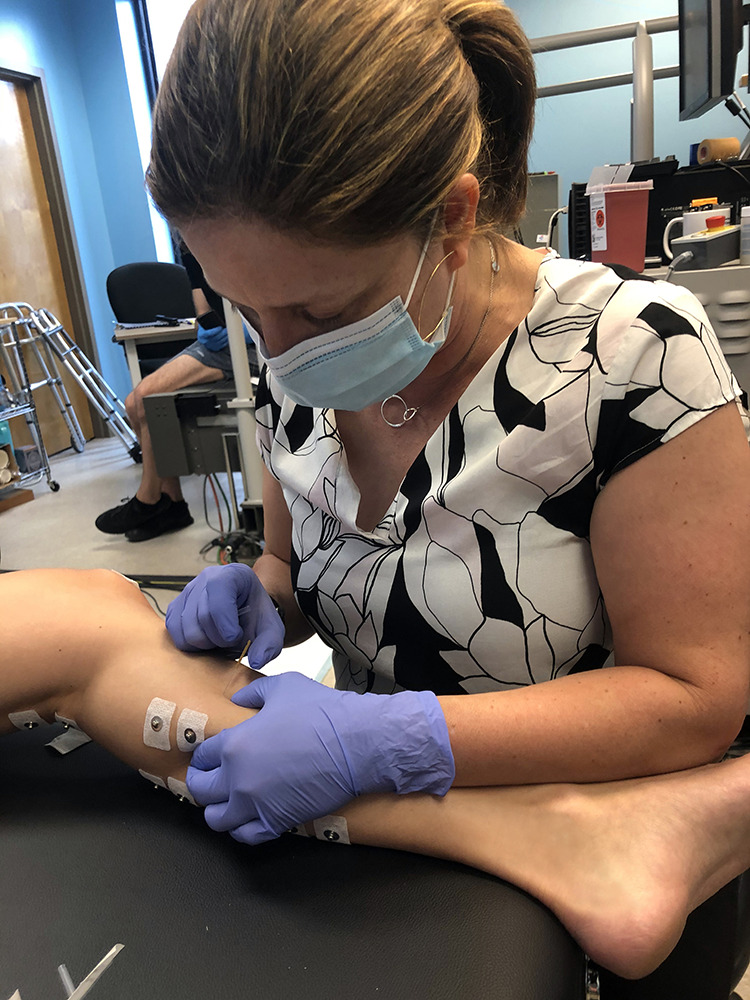
Deep dry needling (DDN) setup. The participant lies supine on a treatment table with the studied leg in a slightly flexed and externally rotated position. DDN is administered at latent trigger point (TrP) until it is eradicated within participant’s tolerance. A typical DDN bout takes 3–5 min in total.

In this study, DDN was administered as in the common clinical practice (4, 6, 8, 14–16, 33), and its procedures are briefly described here. During DDN administration, the participant was supine on a treatment table with the studied leg in a slightly flexed and externally rotated position for comfort and access to MG (Fig. 2). First, the TrPs, localized hypersensitive spots in palpable taut bands of the targeted muscle (6, 71) (i.e., MG in the present study), were identified with pincer palpation. Then, after the skin area over TrPs was cleaned with alcohol swabs, a disposable stainless steel acupuncture needle was inserted into the skin near one of the identified TrPs, penetrating ∼15–20 mm to the general depth of MG TrPs, until a LTR was elicited. Once the LTR was confirmed, the needle was moved up and down (∼4–5 mm vertically) with small piston motions (approximately at 1 Hz for 25–30 s), with no rotation at the TrP (12, 14, 15). This procedure was repeated at other TrPs in the same local skin region as needed until all the LTRs were eradicated. After removal of the needle, the needled area was rubbed to prevent bleeding and bruising. After DDN, each participant completed five repetitions of each of the following: ankle plantarflexion/dorsiflexion passive ROM and submaximal concentric and eccentric contractions of the plantarflexors as recommended by Diciolla et al. (72).

### Data Analysis

#### H_max_ and M_max_.

For each of the triceps surae muscles, the H_max_ and M_max_ were obtained from the recruitment curve measured during standing. Typically, the H reflex was measured over the 30–45 ms poststimulus period and the M wave over the 6–21 ms poststimulus period for the soleus; for the MG and LG, the time windows of analysis were slightly shifted: 28–42 ms post stimulus for the H reflex and 5–20 ms post stimulus for the M wave (67, 68). For each participant, the amplitudes of H reflex and M wave were calculated as the average peak-to-peak values for each stimulus intensity, and the maximum values among those averaged H reflex and M wave values were defined as the H_max_ and M_max_, respectively. Based on the H_max_ and M_max_ values, the H_max_/M_max_ ratio was also calculated. Similarly, for the TA, the M wave to CPn stimulation was measured (typically) over the 3.5–20 ms poststimulus period and its maximum value was defined as the M_max_.

#### Reciprocal inhibition.

To quantify the amount of inhibition of the ongoing EMG activity produced by the antagonist muscle nerve stimulation (i.e., CPn stimulation), first, the triceps surae EMG was rectified. Then, the mean rectified EMG amplitude for a 7- to 10-ms period including the peak suppression (58, 66) was calculated for the CPn stimulation trials, in which the TA M wave amplitude was ∼50% M_max_. When the period of inhibition appeared to be longer than 20 ms, the inhibition was measured over the first 10 ms. Because it is measured over a period of time, the inhibition may contain disynaptic and other short-latency reflex components. Thus, we refer to this as (short latency) reciprocal inhibition, without specifying neural pathways that may be involved (58, 66). For each of the triceps surae, the amount of inhibition was calculated as the difference in the EMG amplitude between the above-specified 7- to 10-ms period and a 50-ms prestimulus period (i.e., background EMG period), and expressed in percentage of background EMG.

#### Statistical analysis.

To assess the stability of experimental and EMG measurement conditions across multiple time points, a linear mixed model (LMM) analysis (time point × participant) was applied to the background (i.e., prestimulus) EMG, M_max_ of the triceps surae and TA, and H_max_ and H_max_/M_max_ ratio of the triceps surae, measured before, immediately after, 90 min after, and 72 h after DDN. To assess the effects of DDN, the LMM analysis (time point × participant) was applied to the H_max_, M_max_, and reciprocal inhibition of the triceps surae and ankle ROM, measured across the above-described four time points. When appropriate, Fisher’s least significant difference (LSD) test was used post hoc to assess differences between measurement time points. Where appropriate, additional correlation analysis was performed among the measures on which the effects of DDN were detected. All statistical analyses were performed with IBM SPSS version 28, and α-level was set at 0.05.

## RESULTS

### Background EMG and M_max_

There were no significant changes in the background EMG across the four measurement time points during the triceps surae H reflex and M wave measurements (i.e., PTn stimulation): soleus (*F*_3,47.8_ = 0.18, *P* = 0.913); TA (*F*_3,47.5_ = 0.94, *P* = 0.427); MG (*F*_3,45.3_ = 2.73, *P* = 0.06); and LG (*F*_3,45.3_ = 0.55, *P* = 0.65). Similarly, no significant changes in the background EMG across the four time points were found during the measurements of triceps reciprocal inhibition (i.e., CPn stimulation): soleus (*F*_3,44.9_ = 0.24, *P* = 0.08); TA (*F*_3,40.8_ = 0.81 *P* = 0.50); MG (*F*_3,37.7_ = 0.73, *P* = 0.54); and LG (*F*_3,42.0_ = 0.68, *P* = 0.57). Thus, with no significant change in the background EMG levels, we could reasonably assume that any changes in H reflex or reciprocal inhibition would be attributable to the DDN intervention, as long as the M_max_ values also remained stable across the measurements (73, 74).

**Figure 3.**
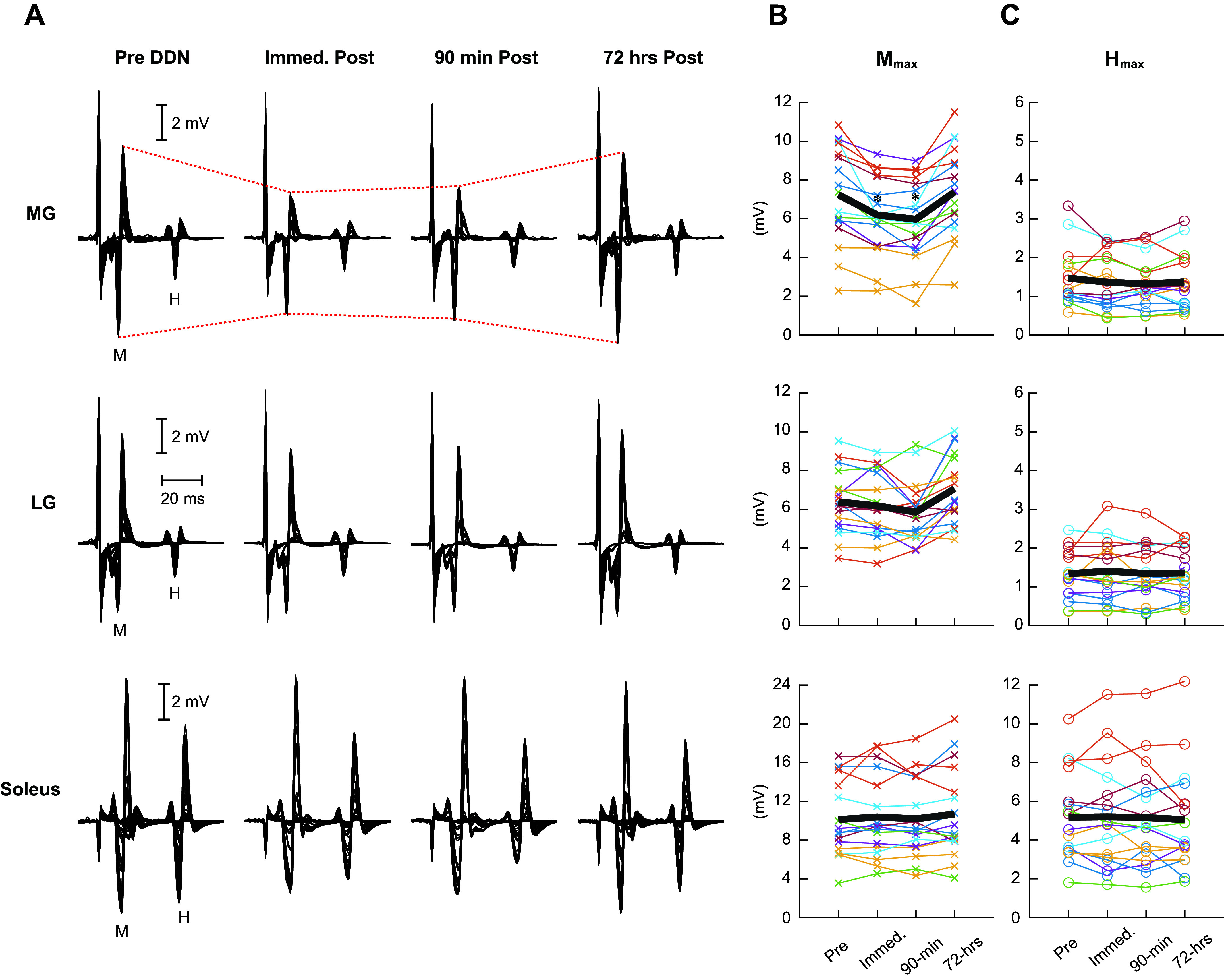
Triceps surae M wave and H reflex before and immediately, 90 min, and 72 h after deep dry needling (DDN). *A*: examples of peristimulus electromyographic (EMG) sweeps for the H reflex/M wave recruitment curve measurements in the medial gastrocnemius (MG, *top*), lateral gastrocnemius (LG, *middle*), and soleus (*bottom*) in a single participant. *B*: the group mean (in black) and individual participants’ maximum M wave (M_max_) amplitudes (in multiple colors) at 4 measurement time points are shown. From *top* to *bottom*, MG, LG, and soleus M_max_ measurements. *C*: the group mean (in black) and individual participants’ maximum H reflex (H_max_) amplitudes at 4 measurement time points are shown. An asterisk near the group mean line indicates a statistically significant difference from the pre-DDN measurement (*P* < 0.05 by a linear mixed model analysis and Fisher’s least significant difference test).

LMM analysis revealed a significant effect of time points for the MG M_max_ (*F*_3,47.3_ = 20.49, *P* < 0.001); MG M_max_ was significantly decreased from pre (7.2 ± 2.4 mV, mean ± SD) to immediate post (6.2 ± 2.0 mV) (*P* < 0.001, Fisher’s LSD as post hoc) and from pre to 90 min post (6.0 ± 2.1 mV, *P* < 0.001) and returned to pre-DDN level at 72 h post (7.4 ± 2.3 mV, *P* = 0.70) (Fig. 3). LMM analysis also indicated an effect of time points for the LG M_max_ (*F*_3,46.9_ = 8.73, *P* < 0.001); however, the values at immediate (6.2 ± 1.7 mV, *P* = 0.4), 90 min post (5.9 ± 1.5 mV, *P* = 0.11), and 72 h post (7.1 ± 1.8 mV, *P* = 0.08) were not significantly different from pre-DDN (6.4 ± 1.6 mV) measurement. For the soleus M_max_, LMM analysis did not reveal a significant effect of time point (*F*_3,47.8_ = 1.07, *P* = 0.37). Thus, for the soleus and LG, with no significant changes in M_max_, we could reasonably assume that any changes in H reflex or reciprocal inhibition would be attributable to the DDN intervention. For the MG, with changes in M_max_ in response to DDN, no further statistical assessment was performed on the H reflex or reciprocal inhibition.

### Effects of DDN on the H_max_

No systematic changes were observed for the triceps H_max_ (Fig. 3). LG H_max_ was 1.3 ± 0.6 mV (mean ± SD) at pre, 1.4 ± 0.7 mV at immediate post, 1.4 ± 0.7 mV at 90 min post, and 1.4 ± 0.6 mV at 72 h post. Soleus H_max_ was 5.2 ± 2.2 mV at pre, 5.2 ± 2.6 mV at immediate post, 5.2 ± 2.5 mV at 90 min post, and 5.1 ± 2.6 mV at 72 h post. LMM analysis revealed no significant effect of time points for the H_max_ in the LG (*F*_3,48.4_ = 0.45, *P* = 0.72) or in the soleus (*F*_3,48.1_ = 0.11, *P* = 0.95). When the H_max_ was expressed as the H_max_/M_max_ ratio, the effect of time points remained insignificant for the soleus (*F*_3,48.7_ = 0.69, *P* = 0.57); soleus H_max_/M_max_ ratio was 0.52 ± 0.11 (mean ± SD) at pre, 0.51 ± 0.17 at immediate post, 0.50 ± 0.14 at 90 min post, and 0.48 ± 0.13 at 72 h post. LMM indicated a significant effect of time point for LG H_max_/M_max_ ratio (*F*_3,48.5_ = 3.68, *P* = 0.02); however, no significant differences were detected through post hoc analysis. LG H_max_/M_max_ ratio was 0.23 ± 0.13 (mean ± SD) at pre, 0.25 ± 0.15 at immediate post, 0.24 ± 0.13 at 90 min post, and 0.20 ± 0.10 at 72 h post. Note that although we did not perform statistical analysis on the MG H_max_ or H_max_/M_max_ ratio (for significant changes observed in the MG M_max_ after DDN, as described above), individual participants’ H_max_ values that were measured are displayed in Fig. 3*C*.

### Effects of DDN on Reciprocal Inhibition

**Figure 4.**
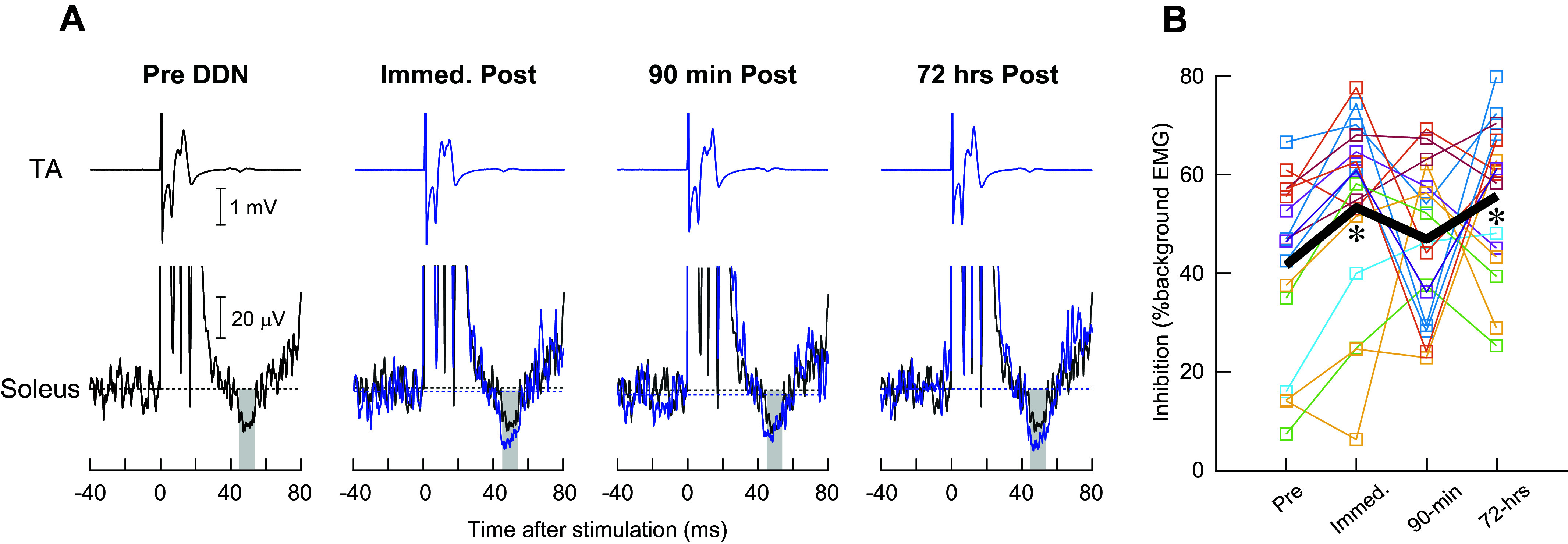
Reciprocal inhibition of the soleus elicited by common peroneal nerve (CPn) stimulation before and immediately, 90 min, and 72 h after deep dry needling (DDN). *A*: examples of tibialis anterior (TA) and (rectified) soleus electromyographic (EMG) sweeps during CPn stimulation near the TA 50% maximum M wave (M_max_) level in a single participant. On the 3 soleus panels for post-DDN measurements, the pre-DDN sweep (in blue) is superimposed for comparison. A gray highlighted window indicates a time window used for calculating the amount of inhibition. Twenty sweeps are averaged together for each sweep. *B*: amount of reciprocal inhibition of the soleus ongoing EMG activity is expressed as %background (i.e., prestimulus) EMG. The group mean (in black) and values from individual participants (in multiple colors) are shown. Asterisks near the group mean line indicate significant increases in inhibition from pre DDN at 2 time points, immediately and 72 h after DDN (*P* < 0.05 by a linear mixed model analysis and Fisher’s least significant difference test).

Effect of time points on reciprocal inhibition of the LG was not significant (*F*_3,43.8_ = 0.20, *P* = 0.90); the amount of LG inhibition was 17.8 ± 21.3% (%background EMG) before DDN and 18.2 ± 25.2%, 18.7 ± 21.3%, and 23.4 ± 28.0% immediately, 90 min, and 72 h post DDN, respectively. In contrast, reciprocal inhibition of the soleus was increased after DDN (Fig. 4). LMM analysis indicated a significant effect of time points (*F*_3,41.9_ = 3.42, *P* = 0.04): soleus inhibition increased from 41.1 ± 18.2% pre DDN to 53.3 ± 19.3% (*P* = 0.03) at immediate post, back down to 46.9 ± 15.1% (*P* = 0.33) at 90 min post, and then up again to 55.7 ± 15.3% at 72 h post DDN (*P* = 0.02) (Fig. 4).

### Effects of DDN on the Ankle Passive ROM

**Figure 5.**
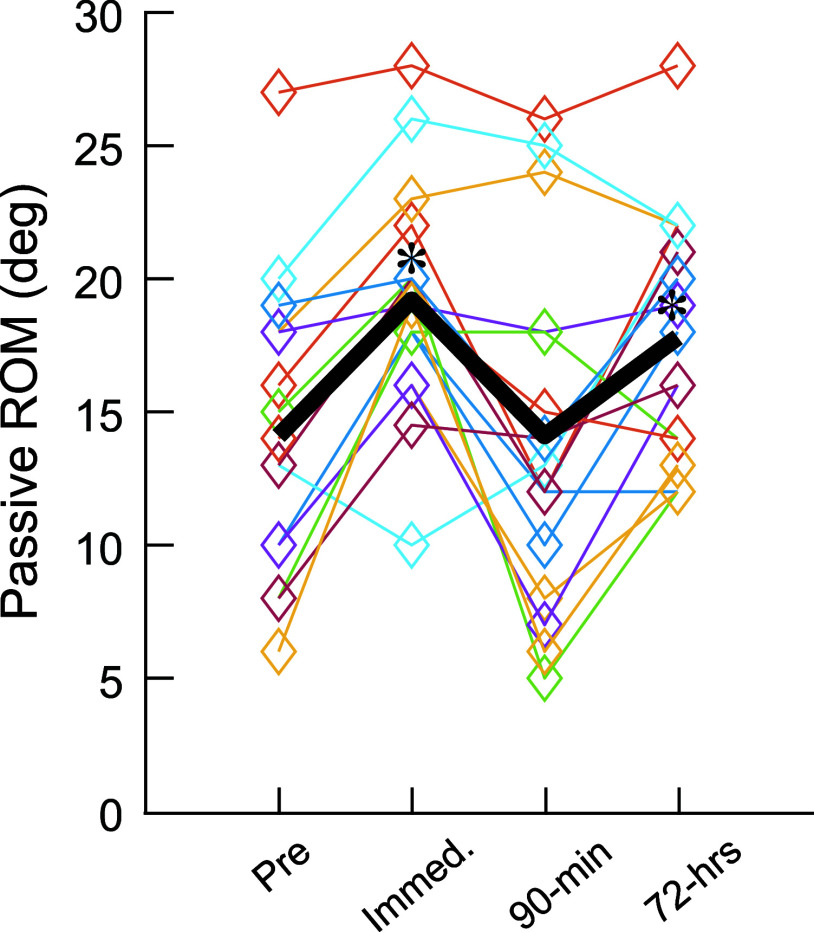
Passive ankle range of motion (ROM) before and immediately, 90 min, and 72 h after deep dry needling (DDN). The group mean (in black) and values from individual participants (in multiple colors) are shown. Asterisks near the group mean line indicate significant increases in the ankle ROM from pre DDN at 2 time points, immediately and 72 h after DDN (*P* < 0.05 by a linear mixed model analysis and Fisher’s least significant difference test).

Effect of time points on ROM was significant (*F*_3,45.8_ = 15.34, *P* < 0.001); the ankle DF ROM was larger at immediate post (19.2 ± 4.1°, *P* < 0.001) than pre DDN (14.1 ± 5.3°) but not at 90 min post (14.1 ± 6.2°, *P* = 0.98) or 72 h post (17.8 ± 4.5°, *P* = 0.02) (Fig. 5).

Since the analyses of ROM and reciprocal inhibition indicated immediate post and 72 h post DDN as potential key time points, the correlation between the changes in reciprocal inhibition and the changes in ROM (i.e., changes from the pre-DDN values) was assessed at immediate post and 72 h post DDN. Correlation coefficient (i.e., *r* value) was insignificant: 0.03 at immediate post and 0.19 at 72 h post DDN for the soleus and −0.23 at immediate post and 0.03 at 72 h post DDN for the LG.

## DISCUSSION

In this study we examined the effects of one bout of DDN at MG latent TrPs on inhibitory and excitatory spinal reflexes in persons without known neurological conditions. The study found that the MG M_max_ amplitude was decreased immediately and 90 min post DDN but returned to its pre-DDN level by 72 h post. Such effects were specific to the treated MG: M_max_ amplitude was not decreased after DDN in the LG, soleus, or TA. For the spinal reflexes, neither LG nor soleus H_max_ changed after DDN, whereas reciprocal inhibition of the soleus was increased at immediate and 72 h post DDN. Passive DF ROM was increased at immediate and 72 h post DDN. Below we discuss potential mechanisms of DDN-induced spinal plasticity and therapeutic implications of TrP DDN for neurorehabilitation after stroke and other neurological injuries.

### Changes in the MG but Not in the LG or Soleus M_max_

In the present study, we observed that the M_max_ became smaller in MG (but not in LG, soleus, or TA) immediately and 90 min after DDN and then it returned to its pre-DDN level 72 h post (see Fig. 3). Since the triceps surae M_max_ becomes smaller when the ankle is in more dorsiflexed positions in sitting (75) or standing (76), one might suspect that the observed temporary reduction of MG M_max_ could be due to a temporary change in the ankle posture during standing posture. This is unlikely, however, because the M_max_ decreased only in MG and not in LG or soleus, both of which would have been affected similarly if there were changes in ankle joint posture. Thus, it is conceivable that the observed MG M_max_ decrease was likely from mechanical actions of DDN [i.e., microinjuries of muscle fibers and nerve axons near neuromuscular junctions (77)] applicable only to the muscle that was treated. The muscle-specific and temporary reduction and recovery of M_max_ are in line with Domingo et al. (77), in which the timelines of muscular degeneration and regeneration and end plate reestablishment and reinnervation after minor muscle and nerve injuries from dry needling were examined. Recovery of motor end plate structure and function from injuries was observed on the third day after needling (77). These findings support that neuromuscular microinjuries caused by DDN of MTrPs are temporary and that motoneuron connection to the treated muscle can be restored by *day 3* post DDN. Generalizability of these muscle-specific reduction and recovery of M_max_ to other leg muscles or upper limb muscles is yet to be determined through future studies.

### Effects of DDN of MG TrPs on Spinal Reflexes

In the present study, we did not observe changes in the LG or soleus H_max_ after DDN. Similar findings have been reported with DDN in soleus (30) and in the fibularis longus muscle (32). Altogether, these suggest that a single bout of DDN of MTrPs does not change the excitability of an excitatory reflex pathway, the H reflex pathway, in lower leg muscles.

In contrast, reciprocal inhibition of the soleus was increased immediately and 72 h post DDN (and to a lesser extent at 90 min post). Since the soleus was not the muscle treated by DDN, the mechanism of soleus inhibition increase would not be in the muscle but in the spinal neurophysiology: it would be from the increased excitability of inhibitory interneurons that receive input from the TA spindle afferents (known as Ia inhibitory interneurons) (57, 60, 78, 79) and/or from reduced presynaptic inhibition at Ia (of CPn)-Ia inhibitory interneuron synapse (79, 80). Excitability of Ia inhibitory interneurons can be modulated by synaptic input from Renshaw cells (81) and other spinal interneurons that receive input from sensory afferents [e.g., cutaneous afferents (82), Golgi tendon organ afferents (83, 84)]. It is also regulated by descending corticospinal drive (85–91); that is, increasing corticospinal input to Ia inhibitory interneurons increases those interneurons’ excitability and thereby increases reciprocal inhibition (90, 91). Efficacy of Ia-Ia inhibitory interneuron connection for reciprocal inhibition can also be modulated presynaptically by descending drive (86, 92) and/or spinal mechanisms (84, 86). Whether, how, and to what extent any (or all) of these possible mechanisms are involved in increased soleus inhibition after DDN is currently unknown. Since there is a study reporting a reduction of active motor threshold for the infraspinatus motor evoked potential 24 h after DDN in individuals with chronic shoulder pain (31), a temporary increase in corticospinal excitability after DDN might be a partial cause of presently observed increases in soleus reciprocal inhibition. Note that it is quite possible that the mechanisms responsible for the inhibition increase at 72 h post DDN are different from those for the inhibition immediate post DDN. For instance, soleus inhibition increase at immediate post might be largely driven by acute increases in corticospinal excitability (31), whereas the inhibition increase at 72 h post DDN might reflect longer-lasting plasticity in neural pathways involved in reciprocal inhibition and/or adaptive plasticity in response to some subtle changes in leg motion (e.g., changes in ankle joint range of motion) as a result of DDN (see Fig. 5).

Although mechanistic details of how reciprocal inhibition of the soleus becomes enhanced after latent TrP DDN are yet to be investigated, the present findings in spinal reflex measurements suggest that DDN does not simply increase or decrease spinal excitability. That is, there was no uniform increase or decrease in both excitatory and inhibitory reflex amplitudes after DDN. Dissociation in presence/absence and time course of reflex changes between an excitatory and an inhibitory reflex supports the view that a single bout of DDN of latent TrP produces complex neurophysiological effects at the spinal level.

### Effects of MG TrPs DDN on Ankle ROM

In this study we observed increases in passive ankle DF ROM at immediate and 72 h post DDN. The immediate effect of DDN on passive ROM was not surprising, as there have been similar reports made by others (93–95). Cruz-Montecinos et al. (94) found an increase in active weight-bearing DF ROM after dry needling of the MG when there was an observable twitch response. Grieve et al. (95) found 4° increase in active DF ROM in recreational runners after manual treatment of triceps surae latent TrPs. Benito-de-Pedro et al. (93) treated latent TrPs with DDN and ischemic compression in triathletes and found 4–5° increased ankle DF ROM with both interventions. Yet there have also been several other studies that found no significant changes in ROM after a single bout of DDN (13, 96, 97). Perez-Bellmunt et al. (13) reported increased relaxation and decreased muscle tone after DDN of the gastrocnemius but no significant changes in DF ROM. Some of these seemingly conflicting findings on DF ROM could be due to methodological differences among studies [e.g., weight-bearing (13, 96) or participants were symptomatic (97)]. Since DDN is one of the methods to treat latent TrPs and application of other methods also increased DF ROM (93, 95), as it stands, it is not clear whether the present observation of increased passive DF ROM at immediate post DDN was due to DDN and not from treating latent TrPs. Clearly, further investigations would be needed to confirm the immediate effects of latent TrP DDN on ankle ROM.

One of the interesting observations from this study is that passive DF ROM that was increased at immediate post decreased at 90 min post and then increased again at 72 h post DDN (see Fig. 5). Previously, Kelly et al. (98) reported immediate reduction of local muscle stiffness after DDN in the gastrocnemius, which would then be followed by increased stiffness at 24 h post. The timeline of increased muscle stiffness by Kelly et al. (98) appears to match the timeline of inflammatory reactions observed in the treated muscle by Domingo et al. (77). Taken together, the rapid increase and decrease in DF ROM that were observed over the first 90 min post DDN may partially be explained by these transient changes in local muscle stiffness and DDN-caused local inflammatory reactions. By 72 h post inflammatory reactions in the muscle would be subsided and end plate reinnervation at neuromuscular junction would be completed (77); at that point, in no longer stiff muscle with restored neuromuscular connections, plasticity that has been happening in the spinal cord may become detectable in measures such as ROM and reflexes that are readily relatable to the state and function of the treated muscle.

As indicated in the present correlation analysis between the extent of reciprocal inhibition changes and the extent of ROM changes, increases in reciprocal inhibition of the soleus would not explain increases in ROM at immediate post or 72 h post DDN, at least in a simple immediate term. However, this does not completely rule out the possibility of an indirect link between reciprocal inhibition and ROM change and/or reciprocal inhibition being one of the multiple factors involved in ROM change. At early (i.e., immediate post) and/or late (i.e., 72 h post) time points after DDN, interneurons with broad neural connections within the spinal cord, such as Renshaw cells (99–101), might be involved in the observed ROM increases. Renshaw cells could be excited by group III and IV afferents (102) that are likely activated during latent TrP DDN (4, 26). Excitation of Renshaw cells could limit firing of motoneurons whose muscles are stretched (during passive ROM testing) and firing of their synergists’ motoneurons through recurrent inhibition (103, 104). Other spinal mechanisms [e.g., presynaptic inhibition of Ia excitatory reflexes (65, 105–108)] may also be involved, but how they may be linked to mechanical actions of DDN cannot be adequately speculated from the present study.

### Study Limitations and Functional Implications

In this study we aimed to examine the effects of DDN of latent TrPs on inhibitory and excitatory spinal reflex pathways. Since there were no prior systematic reflex studies with DDN, in this early stage of investigation we opted to treat all participants with the real DDN of latent TrP; none received sham or control treatment. Thus, the extent of DDN effect reported here would need to be confirmed through future investigations. Initial findings from this study support the worthiness of further investigation of spinal plasticity associated with DDN in more refined designs (e.g., with control and/or sham treatment groups and more measurement time points).

In interpreting the present results for functional implications, a couple of issues are worthy of discussion. First, since none of the present participants had neurological deficits or sensorimotor impairments, the observed extent and time course of changes in EMG measures and ROM may not be immediately transferable to clinical populations, such as individuals after spinal cord injury or stroke. It has been shown that neural plasticity induced by neuromodulatory interventions could appear differently across different populations (69, 109–111). Second, DDN of latent TrPs would reduce the neural function at/near the treated site [e.g., Domingo et al. (77) and Fig. 3]. The effects are specific to the treated muscle and temporary, and the function of damaged neuromuscular junctions and end plates would be restored in ∼72 h. These lead us to the following potential implications. *1*) Considering the specificity of DDN effects on the M_max_, treating the muscle of interest directly would be a preferred method for most clinical applications (i.e., treating spastic muscles). *2*) TrP DDN could be viewed as a method to disable specific neuromuscular connections temporarily, in contrast to a method of permanency [e.g., botulinum toxin (112, 113)]. TrP DDN might create a window of opportunity for clinicians in administering motor retraining activities that require the treated muscle to be more flaccid (i.e., immediate to 90 min post DDN time window) or activities that benefit from increased ROM (i.e., 72 h post DDN). *3*) As mentioned above, treating TrPs with methods other than DDN also produces an immediate increase of ROM (93, 95); however, the time needed for posttreatment muscular recovery could be variable (77) if those methods temporarily disable neuromuscular connections. Currently the comparability of DDN and other methods to treat TrPs in neurophysiological and functional effects is not established, and thus one should not speculate the clinical utility of DDN based on those other methods of treating TrPs.

### Conclusions

In this study we found that the M_max_ amplitude was decreased immediate and 90 min post DDN but returned to its pre-DDN level by 72 h post in the DDN-treated MG but not in the LG, soleus, or TA. Whereas the H_max_ did not change after DDN, reciprocal inhibition of the soleus was increased after DDN. DDN appears to cause temporary and specific microinjuries to the muscle and nerve of the treated muscle, whereas its neurophysiological (plasticity) effects are not limited to the muscle treated. Interestingly, the time course of DF ROM changes was similar to that of reciprocal inhibition: both were increased at immediate post and 72 h post (Fig. 4 and Fig. 5). To our knowledge, there is no known causal relationship between reciprocal inhibition and ROM; however, a possibility that the same causal factor could be involved in increasing ROM and reciprocal inhibition remains. Overall, how excitatory and inhibitory reflexes responded differently to DDN and unique time course of changes in soleus inhibition and ankle ROM may suggest complex neurophysiological effects of DDN at the spinal level. Whether and to what extent the present findings in individuals with latent TrP are applicable to those with active TrP and whether similar findings can be translated to persons with spasticity due to neurological disorders are yet to be examined in future studies.

## DATA AVAILABILITY

The datasets generated and/or analyzed during the current study are available from the corresponding author on reasonable request.

## GRANTS

This work was supported in part by the Eunice Kennedy Shriver National Institute of Child Health and Human Development (P2C HD086844 to S. Kautz), the National Institute of General Medical Sciences (P20 GM109040 to S. Kautz), and the Doscher Neurorehabilitation Research Program.

## DISCLOSURES

No conflicts of interest, financial or otherwise, are declared by the authors.

## AUTHOR CONTRIBUTIONS

G.S., A.M.P., J.M.D., B.H.S.D., and A.K.T. conceived and designed research; G.S., A.M.P., B.H.S.D., and A.K.T. performed experiments; A.M.P., B.H.S.D., and A.K.T. analyzed data; G.S., A.M.P., J.M.D., B.H.S.D., and A.K.T. interpreted results of experiments; B.H.S.D. and A.K.T. prepared figures; G.S., A.M.P., B.H.S.D., and A.K.T. drafted manuscript; G.S., A.M.P., J.M.D., B.H.S.D., and A.K.T. edited and revised manuscript; G.S., A.M.P., J.M.D., B.H.S.D., and A.K.T. approved final version of manuscript.
